# Tribotechnical Properties of Sintered Antifriction Aluminum-Based Composite under Dry Friction against Steel

**DOI:** 10.3390/ma15010180

**Published:** 2021-12-27

**Authors:** Nikolay M. Rusin, Alexander L. Skorentsev, Maksim G. Krinitcyn, Andrey I. Dmitriev

**Affiliations:** Institute of Strength Physics and Materials Science of Siberian Branch Russian Academy of Sciences (ISPMS SB RAS), 2/4, pr. Akademicheskii, 634055 Tomsk, Russia; rusinnm@mail.ru (N.M.R.); krinmax@gmail.com (M.G.K.); dmitr@ispms.ru (A.I.D.)

**Keywords:** antifriction aluminum alloy, sintering, dry friction, wear mechanism

## Abstract

The disadvantage of antifriction Al–Sn alloys with high tin content is their low bearing capacity. To improve this property, the aluminum matrix of the alloys was alloyed with zinc. The powder of Al–10Zn alloy was blended with the powder of pure tin in the proportion of 40/60 (wt.%). The resulting mixture of the powders was compacted in briquettes and sintered in a vacuum furnace. The sintered briquettes were subjected to subsequent pressing in the closed press mold at an elevated temperature. After this processing, the yield strength of the sintered (Al–10Zn)–40Sn composite was 1.6 times higher than that of the two-phase Al–40Sn one. The tribological tests of the composites were carried out according to the pin-on-disk scheme without lubrication at pressures of 1–5 MPa. It was established that the (Al–10Zn)–40Sn composite has higher wear resistance compared with the Al–40Sn one. However, this advantage becomes insignificant with an increase in the pressure. It was found that the main wear mechanism of the investigated composites under the dry friction process is a delamination of their highly deformed matrix grains.

## 1. Introduction

Increasing the service life of friction units in machines and mechanisms is one of the most important problems of modern materials science and engineering [1,2,3]. Alloys of Al–Sn system, unlike other aluminum-based alloys, have a good resistance to seizure, especially at high content of the soft phase [2,3,4,5,6,7,8,9]. The hardness and bearing capacity of Al–Sn alloys are decreased with an increase in tin content because Sn has a low melting point and is not strengthened under deformation processing. As a result of the low mechanical properties, the alloys with high Sn content are usually used in sliding bearings as a thin coating on hard inserts.

The manufacturing technology of multilayer bearing inserts is relatively complicated and expensive. Hence, such multilayer inserts should be replaced by monometallic ones if it is possible. For this purpose, the strength of the Al–Sn alloys should be increased by alloying of the alloys with hard particles or some materials that form a solid solution with Al [10,11,12]. Simultaneously, it is necessary to avoid dissolution of these strengthening additives in Sn inclusions in order to maintain a high level of ductility and lubricating ability of the soft phase. For this reason, the list of the additives is limited [13,14].

In order to increase the strength of aluminum alloys, they are often alloyed with silicon or hard ceramic particles [7,8,11,15]. However, their surface is poorly wetted by liquid tin, and weak interphase boundaries are formed in the alloys during the melt crystallization. The alloying of Al–Sn alloys with copper, which forms a solid solution with aluminum, does not lead to a noticeable increase in their wear resistance. During the crystallization, copper forms hard compounds with low-melting tin. As a result, tin inclusions are hardened, and their lubricating properties are sharply decreased [16,17].

**Figure 1:**
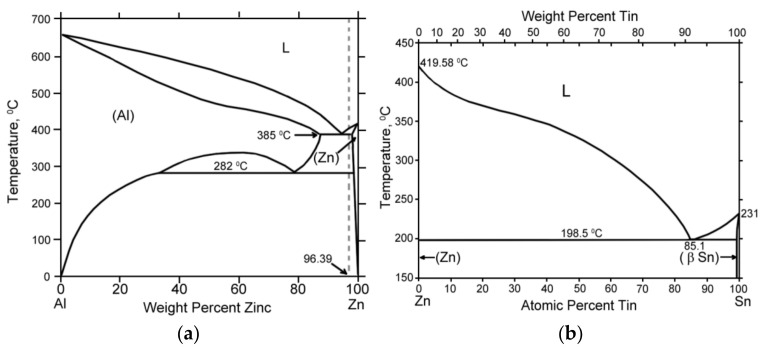
Phase diagrams of Al–Zn (**a**) and Zn–Sn (**b**) binary alloys.

According to the phase diagram of Al-Zn [18], zinc is one of the promising alloying elements because it is well dissolved in aluminum and almost not dissolved in solid tin [19] (Figure 1). Therefore, Zn cannot strongly affect the plasticity of Sn inclusions. Moreover, the amount of zinc in tin inclusions can be significantly decreased if the Al–Zn–Sn composite material (CM) is prepared by sintering of Al-Zn alloy and tin powders instead of industrial casting methods. The sintering of such a mixture allows preparing the CM with a prehardened aluminum matrix without a stage of complete melting of Al and stirring of it with Sn [20,21].

**Figure 2:**
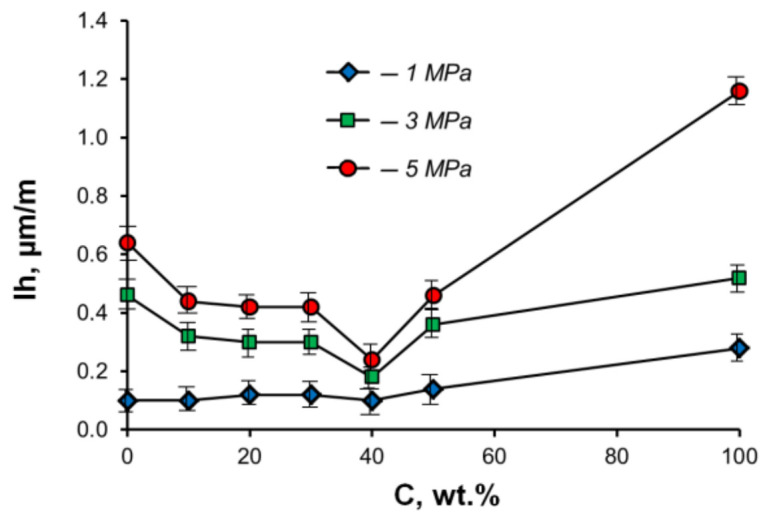
Effect of tin content (*C*) on the wear intensity (*Ih*) of sintered Al–Sn alloys at different pressures under dry friction against steel. Sliding speed is 0.6 m/s.

Another advantage of using the powder metallurgy method for preparing Al–Zn–Sn CM is a tendency of Al powders to percolation and formation of a continuous skeleton, even at a low content. The presence of such a skeleton of the solid powders during the liquid-phase sintering allows an increase of tin content in the sintered samples without a significant separation of the light and heavy phases. It was established that the matrix skeleton in the sintered Al–Sn CM containing up to 40 wt.% of the heavy soft phase has high contiguity. It is important to note that the tin content in industrial cast aluminum alloys is limited to 20 wt.% (10 vol.%) due to disintegration of their aluminum matrix by tin inclusions and a decrease in the load-bearing capacity of the alloys. Dry friction tests of sintered Al–Sn composites against a steel counterbody showed that the samples with about 40 wt.% of tin have highest wear resistance (Figure 2), and this value is higher than that of industrial aluminum alloys [22,23]. It was found that an increase in the tin content up to 40 wt.% also leads to an increase in the wear resistance of aluminum alloys prepared by rapid cooling [6,24].

This work aimed to study the tribological properties of sintered (Al–10Zn)–40Sn composites under dry friction against steel and compare the obtained data with those of sintered Al–40Sn composite in order to identify an effect of the solid solution of Al matrix on the wear resistance of the self-lubricating materials.

## 2. Materials and Methods

The studied CMs were prepared by sintering of a mixture of nitrogen-atomized Al–10Zn alloy and pure tin (PT 2) powders, taken in weight ratio of 3:2, respectively. For comparison, the composite with a pure aluminum matrix and the same tin content was prepared. The sintered briquettes were subjected to subsequent hot pressing (*HP*) in a closed press mold at 200 °C under a pressure (*P*) of about 300 MPa in order to eliminate their residual porosity. The press mold used for the *HP* processing was the same as in the case of compaction of raw briquettes. The density of the raw, sintered, and *HP-ed* briquettes was determined by hydrostatic weighing using analytical balance with an accuracy of 0.0001 g (Standard RF 20018-7). Samples for mechanical and tribological tests of the composites were cut from the sintered and *HP-ed* briquettes. Each test was repeated three times under the same conditions, and each value in the tables is the average one of these three repetitions.

**Figure 3:**
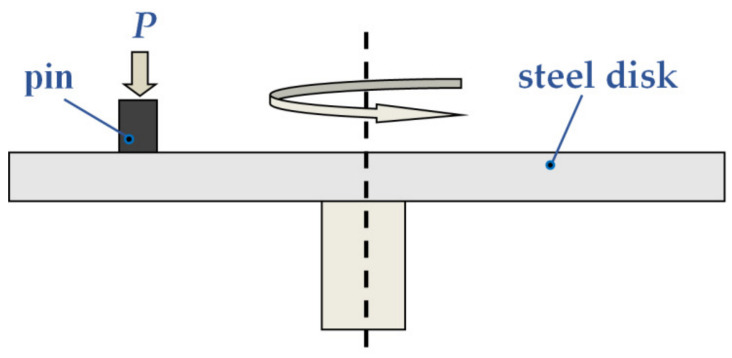
Scheme pin-on-disk of the friction test.

The mechanical properties of the CM were determined by compression test using Walter+BaiAGLFM-125 (Lohningen, Switzerland) universal testing machine (Standard RF 25.503-97). The prismatic samples with the size of 5 × 5 × 10 mm were tested at the compression rate of 0.5 mm/min. The tribological tests were carried out using a tribotester (Tribotechnic, Clichy, France) according to the scheme pin-on-disk without lubrication (Figure 3). This scheme is rarely used in real friction units. However, it has been used by many authors because it is very easy to implement, and the test conditions are easy to reproduce. For this reason, this scheme allows comparing the tribotechnical properties of different materials with the data obtained by various authors. The friction surface of the samples (pins) was 2 × 2 mm. The counterbody (disk) with a hardness of 47 ± 2 HRc was prepared from a hardened structural steel 40H (AISI 5140 steel) (Table 1) that is often used as wear-resistant crankshafts in different machines.

Before the tribological tests, the friction surfaces of the coupled bodies were processed by mechanical grinding with emery paper and subsequent polishing on a cloth smeared with diamond paste having abrasive particles smaller than 1 μm. Then, these surfaces were cleaned with acetone. The sliding speed (*V*) varied from 0.3 to 0.9 m/s, and the pressure on the friction surface (*P*) was 1–5 MPa. The value of friction coefficient (µ) was determined automatically using a built-in computer. The sliding distance was 1000 m for each test.

The linear wear intensity (*Ih*) was used as a characteristic of wear resistance of the CM samples. It was defined during the stage of steady state friction by the following formula: *Ih* = Δ*h*/*L*, where Δ*h* is change in the sample height (μm), and *L* is the sliding distance (m). The value of *Ih* is a convenient characteristic of tribological properties of materials because it allows comparing our results with other ones. For example, if the obtained value of *Ih* is multiplied by the surface area of the sample, we can obtain a volumetric characteristic of its wear intensity, and the subsequent multiplication of the last value by the density leads to obtaining a mass characteristic of the wear intensity. The measurement accuracy of the *Ih* value was 0.02 μm/m.

The structure of the sintered and *HP-ed* CM was investigated using metallographic cross sections, which were prepared according to the above-mentioned method of surface preparation and subjected to subsequent short-term chemical etching in 4% solution of nitric acid in ethanol. After the tribological tests, the sample’s structure in the subsurface layer was studied on sections perpendicular to the friction surface [25]. Optical AXIOVERT-200MAT and scanning electron LEO EVO 50 (Zeiss, Germany) microscopes were used for the structure research. The scanning electron microscopy investigations were performed using secondary electron imaging. The friction track relief was studied using a Zygo NewView 6200 scanning whitelight interference microscope. This equipment was provided by shared use center Nanotech of the Institute of Strength Physics and Materials Science SB RAS (Tomsk, Russia).

## 3. Results

**Figure 4:**
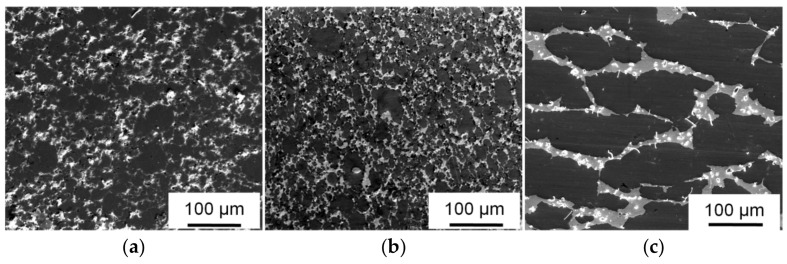
SEM images of the structure of (Al–10Zn)–40Sn (**a**,**b**) and Al–40Sn (**c**) CM; (**a**) after sintering at 600 °C for 1 h; (**b**,**c**) after subsequent *HP* at 200 °C.

The structure of sintered (Al–10Zn)–40Sn and Al–40Sn CMs consists of an Al matrix with uniformly distributed tin inclusions (Figure 4). Simultaneously, the sintered CM contains pores, which significantly decrease their ductility and strength. In order to improve these properties, the sintered briquettes were subjected to *HP* processing in the closed press mold. After this treatment, the macrostructure parameters of the CM are almost unchanged due to the small deformation (Figure 4b). However, the strength of the CM is significantly increased because of elimination of the pores (Table 2). Especially, the *HP* processing has a positive effect on the yield strength (σ_0.2_) of the (Al–10Zn)–40Sn composite, and this value is about 30% higher than that of the composite with pure aluminum matrix due to solid solution hardening of the matrix with zinc. For this reason, further tests were carried out using only the *HP-ed* CM samples.

The results of the tribological tests are shown in Table 3. It can be seen that the wear resistance of the sintered (Al–10Zn)–40Sn composite is lower than that of the Al–40Sn one. However, the opposite tendency was observed after the *HP* processing of the CM. The energy parameter (*P·V*) also has a strong influence on the *Ih* value of these materials. For example, it was found that the increase in *P* leads to a remarkable increase in the *Ih* value regardless of the composition of the aluminum matrix. As a result, the values of *Ih* of the investigated CM are almost equal at the pressure of 5 MPa.

The value of *P* has the same effect on the *Ih* of the CM at the different sliding speeds. However, the dependence of *Ih* on the sliding speed at the constant *P* is more complicated. The value of *Ih* is always decreased with increasing *V* from 0.3 to 0.6 m/s. However, the increase in *V* from 0.6 to 0.9 m/s causes a significant increase in the *Ih* value at the pressure of 5 MPa. The effect of sliding speed on the value of *Ih* is reduced at the low *P* and becomes almost insignificant at *P* = 1 MPa (Table 3). It can be concluded from the above-mentioned results that the optimal sliding speed of the aluminum-based CM with 40 wt.% of Sn is between 0.3 and 0.9 m/s under the dry friction process. The existence of an optimal sliding speed in the case of self-lubricating aluminum-based CM was also found in [26,27].

Elemental composition of the friction surface of the investigated samples, which is an average value of 10 measurements in different points of the smooth and damaged areas, is shown in Table 4. It was established that the initial concentration ratio of the components (Al, Zn, and Sn) is almost unchanged on the friction surface of the CM samples, but this surface is saturated with oxygen and iron during the friction process. The oxygen content on the friction surface is increased at the beginning of the friction test and fast reaches some approximately constant (relative to Al) value. Then, this value varies slightly regardless of the friction test duration and the value of parameter (*P·V*). Iron content on the friction surface, on the contrary, depends on the friction mode and becomes higher with increasing sliding speed. According to [28], this iron is mostly elemental, but about 10% of it is contained in oxides as Fe^2+^.

**Figure 5:**
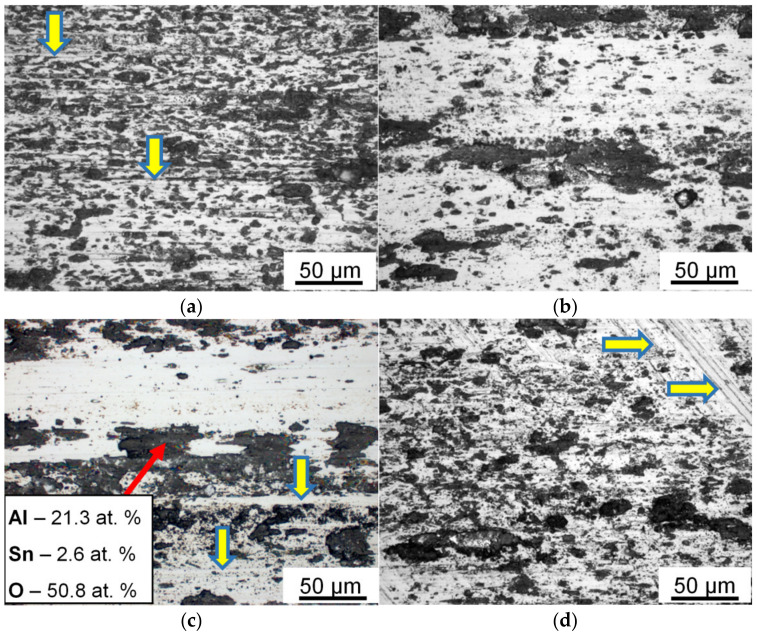
Optical images of the friction track surface on steel counterbody after dry frictional contact with *HP-ed* (Al–10Zn)–40Sn composite sample. *P* = 5 MPa (**a**–**c**) and *P* = 1 MPa (**d**). *V*, m/s: 0.9 (**a**); 0.3 (**b**); 0.6 (**c**,**d**). Traces of the preliminary processing of the counterbody surface are shown by horizontal arrows, and grooves formed on the friction track are shown by vertical arrows.

The elemental composition and relief of the friction track surface on the steel counterbody are also changed. This surface is covered with particles, which are transferred from the sample surface (Figure 5) [22,29]. These particles have an elongated shape in the sliding direction. Their average size decreases with increasing sliding speed at the constant pressure. It becomes higher with increasing *P* at the constant *V* (Figure 5c,d), but a number of the transferred particles and the area of the friction track covered by them are decreased simultaneously.

In any case, the transferred layer is not able to fully protect the counterbody surface from an abrasive action of hard particles. As a result, grooves are formed on the friction track (shown by vertical arrows in Figure 5), and the counterbody surface wears out. The higher the acting pressure, the higher the wear intensity of the counterbody surface. In the case of *P* = 5, the thickness of the worn layer of the friction track is high, and traces of the preliminary processing of the counterbody surface are practically absent. Whereas, at low pressure of 1 MPa, the traces partially remain on the friction track surface, even after a sliding distance of 1000 m (shown by horizontal arrows in Figure 5d).

Iron wear particles move along the friction track for some time. Some part of these particles is captured by damaged areas on the (Al–10Zn)–40Sn sample surface, and another smaller part of them is pressed into the smooth surface areas. The number of such pressed iron particles is increased with increasing the sliding speed because the surface layer of the sample is heated and becomes softer (Table 4). It should be noted that a similar transfer of iron from a hard counterbody to a softer sample was observed during the friction of pure aluminum and antifriction Al-based alloys samples against hardened steel, even in the presence of a relatively thin film of a liquid lubricant [10,30,31,32]. That is, hard abrasive particles are always formed on the friction surface of aluminum-based samples under dry and boundary friction, and they are able to scratch the surface of a steel counterbody.

**Figure 6:**
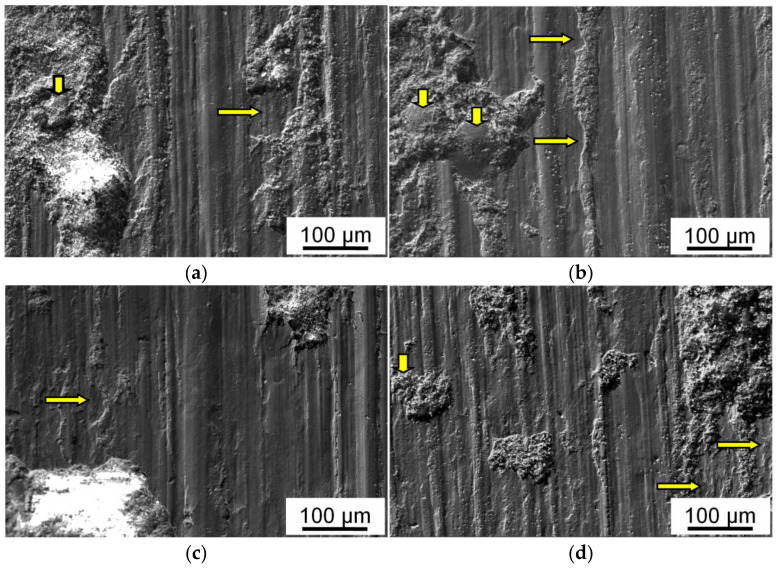
SEM images of the friction surface of *HP-ed* (Al–10Zn)–40Sn composite sample after dry friction against steel at high (*P* = 5 MPa) (**a**–**c**) and low (*P* = 1 MPa) (**d**) pressures. *V*, m/s: 0.9 (**a**); 0.3 (**b**); 0.6 (**c**,**d**). Areas of delamination of wear particles are shown by horizontal arrows, and delaminated particles pressed into the friction surface are shown by vertical arrows.

The friction surface of the tested CM consists of relatively smooth areas, which are intersected by parallel grooves having a low gradient slope and numerous damaged areas (pits) filled with small wear particles (Figure 6). A relief of the friction surface is almost unchanged with the variation of parameter (*P·V*). In addition, there are some recessed areas (shown by horizontal arrows) in the place of delamination of wear particles. Such particles are shifted, compressed between the coupled bodies, and crushed. Then, some of the crushed particles leave the friction track, and the rest of them are adhered to the friction track surface or captured by the damaged areas (shown by vertical arrows).

## 4. Discussion

The uniform distribution of components and phases that are very different in density is a serious problem, especially in the case of large castings and a wide temperature range of the melt crystallization. The powder metallurgy method for preparing such alloys allows a uniform distribution of their components to be achieved without a stirring of the cooling melt. The distribution of the components in sintered materials is set at the stage of the preparation and mixing of powders. During the sintering, their spatial arrangement is maintained by the existence of a solid phase skeleton. In our case, zinc is distributed uniformly in the volume of the sprayed aluminum powders, which, in turn, remain solid during the sintering and limit the movement of liquid tin over the volume of the sample. As a result, the sintered composite has a fine-grained (Al–10Zn) matrix, and tin is distributed in the form of thin interlayers along the grain boundaries (Figure 4a). A small distance between the Sn interlayers should have a favorable effect on the wear resistance of the composite if Sn performs the function of a solid lubricant on the friction surface under dry friction process [33].

However, it turned out that the wear resistance of the sintered composite sample with the fine-grained alloyed aluminum matrix is slightly lower than that of the sample with the coarse-grained unalloyed matrix (Table 3). Whereas, the strength of the tested samples and the rate of their strain hardening are approximately the same (Table 2). Hence, it can be concluded that the features of tin distribution have little effect on mechanical properties and wear resistance of the Al–Sn composites, and tin does not work as a solid lubricant for the friction surface of such materials. Chemical analysis of the friction surface confirms this conclusion because it did not detect an additional amount of tin (Table 4).

It follows from Table 2 that neither the solid solution hardening of the matrix with zinc nor the refinement of its grain structure (Figure 4) leads to a significant increase in strength of the sintered CM. This situation takes place if grains in the CM matrix are weakly involved in a deformation under loading of the CM samples. Tin inclusions in the CM are not hardened under normal conditions due to the high homologous temperature of a deformation and, hence, are places of preferred localization of plastic deformation. Their high content (40% wt.) and continuous net (Figure 4) promote a localization of plastic flow in the soft phase. Pores in the sintered CM are also located at the grain boundaries of the Al matrix and additionally decrease the flow stress of the tin net. As a result, a deformation begins to localize in the tin interlayers at the low flow stress.

The wear intensity of the sintered CM containing pores is increased rapidly with increasing the applied pressure (Table 3). This fact indicates that the sintered CM has a low bearing capacity. Usually, to eliminate pores, sintered materials are deformed by extrusion or other severe plastic deformation methods under high hydrostatical pressure. *HP* of the sintered samples in the closed press mold differs from the above-mentioned processing methods by insignificant deformation of the samples. As a result, after the *HP* processing, the dimensional characteristics of grain structure of the matrix in both the processed CMs are almost unchanged (Figure 4a,b). However, even such a small deformation under the hydrostatical pressure is sufficient to eliminate pores or significantly reduce their volume and improve the quality of the interphase boundaries. The value of σ_0.2_ of (Al–10Zn)–40Sn composite almost doubles as a result of the *HP* processing (Table 2). Its growth is less in the case of the Al–40Sn composite sample because it has nonhardened matrix and lower porosity after sintering. The more significant increase in σ_0.2_ in the case of (Al–10Zn)–40Sn composite sample leads to a remarkable increase in the elastic component of the surface deformation during the friction process. As a result, the wear intensity of the *HP-ed* composite with alloyed aluminum matrix is lower than that of the Al–40Sn one (Table 3).

Nevertheless, the above-mentioned increase in the strength of the investigated CM is insufficient to avoid their significant deformation during the frictional contact against a steel disk. This fact is confirmed by the high concentration of oxygen on the friction surface of the CM samples (Table 4). Since oxygen does not form solid solutions with aluminum and tin, a large amount of it can be present on the friction surface only in the form of oxide films on the new pure surface of aluminum or tin. Such a pure surface is formed during the friction process periodically. That is, under a dry friction process, the surface of the CM samples is subjected to multiple small plastic deformations, which causes cracking of the protective oxide films and their constant reproduction on the pure areas. This type of wear is called an oxidative wear.

**Figure 7:**
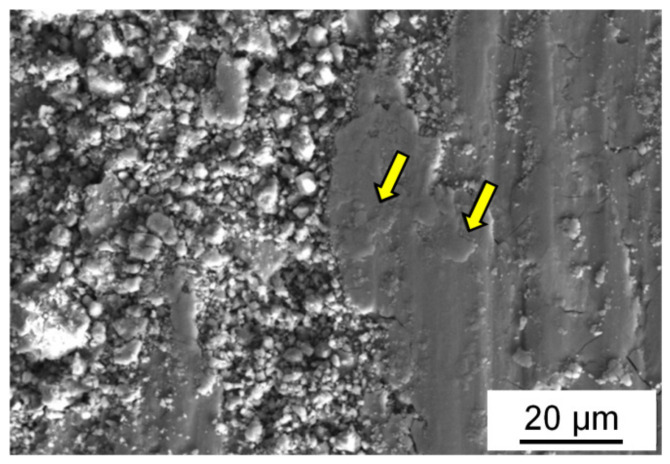
SEM image of the friction surface of *HP-ed* (Al–10Zn)–40Sn composite sample after dry friction against steel. *P* = 5 MPa; *V* = 0.6 m/s. Large delaminated wear particles that are pressed into the sample surface and form grooves on the counterbody surface are shown by arrows.

**Figure 8:**
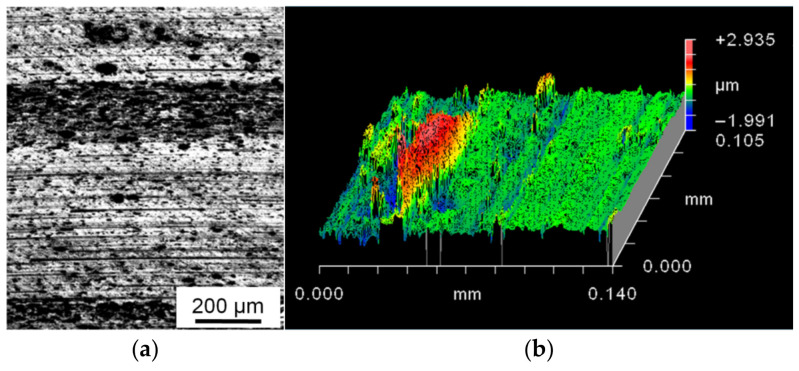
Optical image of the friction track surface on steel counterbody (**a**) and its profile obtained using scanning interference microscope (**b**). *L* = 1000 m, *P* = 1 MPa, *V* = 0.6 m/s.

It can be concluded from images of the friction surface of the (Al–10Zn)–40Sn composite samples (Figure 6 and Figure 7) that their main wear mechanism is the above-mentioned oxidative wear, and its intensity is controlled by processes of fracture and reproduction of the oxide films. The additional pure surface appears as a result of the formation of grooves by moving irregularities of hard counterbody. They penetrate the surface of the CM samples and shift the material in the sliding direction and to the side. Fragments of the oxide films are mixed with a deformed material, and a thin brittle layer is formed on the friction surface. It wears out and determinates the wear intensity of the samples. The formed wear particles have high hardness due to high oxides content in them and scratch the surface of a steel counterbody (Figure 8). As a result, steel shavings are formed, and some of them are transferred to the sample surface and pressed in it or captured by deep pits, which is confirmed by chemical microanalysis of the friction surface of the CM samples (Table 4).

However, it can be seen in Figure 6 and Figure 7 that grooves on the friction surface are wider than hard crushed fragments of the severely deformed upper layer of the samples. The transverse dimension of the grooves (15–30 μm) is equal to that of the particles transferred from the samples surface and adhered to the friction track during the stage of steady state friction (Figure 5 and Figure 8). Particles of the same size were also found on the friction surface of the tested samples (shown by arrows in Figure 6 and Figure 7). It must be assumed that such large particles are formed mainly as a result of delamination of highly deformed matrix grains from the friction surface. The places of such a delamination are shown by horizontal arrows in Figure 6. Hence, the delamination of large particles is a second wear mechanism of the investigated friction pair.

**Figure 9:**
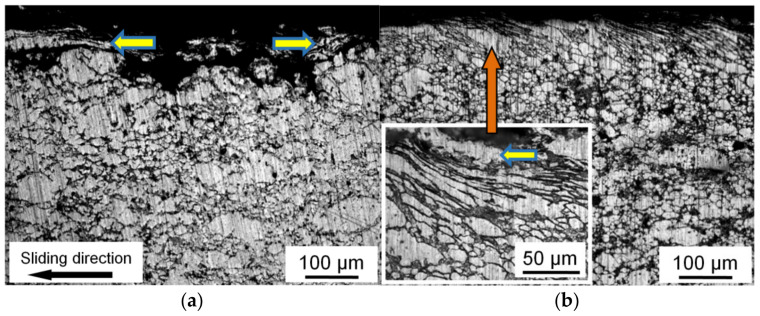
Optical images of the subsurface layer structure of the (Al–10Zn)–40Sn composite sample after dry friction against steel. Condition of the sample: (**a**) sintered, (**b**) subjected to subsequent *HP*. *P* = 5 MPa; *V* = 0.6 m/s. Delaminated wear particles are shown by horizontal arrows.

This mechanism can be seen in more detail in images of the metallographic cross sections perpendicular to the friction surface (Figure 9). The thin upper layer of the CM samples is highly deformed and shifted in the sliding direction. This shift leads a shear deformation of the underlying material of the samples [25]. As a result, the subsurface grains have a shape of elongated flat particles (shown in the inset in Figure 9b) [30,34]. With increasing such a deformation, the thickness of tin interlayers between the subsurface grains is gradually decreased. As a consequence, the resource of their plasticity is exhausted, and a fragment of the highly deformed subsurface layer is delaminated as a large wear particle. Then, a deep pit that is formed in the place of delamination of the large wear particle accumulates smaller wear particles. The thicker a tin interlayer between the matrix grains, the higher the shear deformation of these grains that can be carried out. Therefore, the CM with high Sn content has the highest wear resistance [22].

Pores and areas of poor wetting of the oxidized Al powders by liquid tin are present in the sintered CM [32]. These defects lead to the formation of wear particles under lower deformations and stresses (Figure 9a), and the wear intensity of sintered CM is high (Table 3). The *HP* processing of the sintered CM leads to elimination of the pores and improvement of adhesive bonds between the phases (Figure 4). As a result, the ductility and the value of strain hardening of the *HP-ed* CM are increased (Table 2). This means that the subsurface matrix grains can be subjected to an additional deformation until their separation from the sample surface (Figure 9b), which automatically leads to decreasing the value of *Ih*.

Thus, the mechanism of wear particles formation on the friction surface of the (Al–10Zn)–40Sn composite (and Al–40Sn composite also) during the stage of steady state friction can be described as follows. The surface of the composite sample is subjected to an abrasive action of hard transferred particles located on the friction track surface. As a result, two highly deformed layers are formed under the friction surface: a thin brittle upper layer filled with fragments of oxide films and a lower layer containing deformed flat grains of the matrix [25,31]. The intensity of delamination of the flat grains mainly affects the wear intensity of the aluminum-based CM with tin. Its value is determined by the tendency of the material for localization of plastic shear and, hence, depends on the presence in its structure of such elements as pores, cracks, and other defects that decrease the strength and ductility of the material. Therefore, the subsequent *HP* of the sintered samples is a useful processing because it decreases the number of pores and structural defects at the interphase boundaries and significantly increases the wear resistance of the samples.

The shape, orientation, and size of tin interlayers in the investigated CM are changed together with the surrounding Al grains under a deformation. Moreover, due to the presence of Sn on the Al grain boundaries, the neighboring Al grains can be shifted relative to each other by a higher value than in a case of only cohesive “Al–Al” boundaries between them. As a result, the shape of Al grains in the (Al–10Zn)–40Sn CM can be significantly changed until the fracture of their interphase boundaries. Easier grain boundary sliding leads to the formation of a layered structure with tin interlayers nearly parallel to the sliding direction in the subsurface layer (Figure 9b).

When the plasticity resource of these interlayers is exhausted, a delamination of the highly deformed upper layer of the CM sample takes place. The formed large wear particles located between the hard oxidized friction surface and the steel counterbody are crushed and then captured by the pits or leave the friction track surface. Some of these particles adhere to the friction track surface and form a discrete transferred layer that separates the steel counterbody from the sample surface (Figure 8).

The surface of transferred particles located on the friction track is additionally deformed and oxidized (shown in the inset in Figure 5c). Therefore, the frictional contact between the coupled bodies is actually transformed into contacts between the transferred particles and thin upper surface layer of the sample that is highly deformed and filled with fragments of oxide films. That is, these mechanical contacts are carried out between the chemically inert surfaces. As a result, there are no seizure traces at the bottom of the grooves (Figure 5 and Figure 8), and the contribution of adhesive wear to the total wear of the investigated CM is small.

Thus, delaminations of the highly deformed subsurface grains, and oxidative and abrasive wear are the main wear mechanisms of the CM of Al–Sn system under dry friction against steel. The delamination is carried out by formation of cracks along the tin interlayers due to exhaustion of their plasticity resource. The addition of zinc increases the yield strength of the aluminum matrix and its ability for strain hardening. At the same time, it does not affect the ductility of tin that is determined by thickness of the Sn interlayers and the value of relative shift of the surrounding Al grains under the deformation. Zinc, as well as aluminum, has a high tendency for oxidation. Therefore, a presence of zinc in the matrix grains does not prevent the formation of a hard layer saturated with fragments of oxide films on the friction surface, which prevents an adhesive interaction of the coupled bodies.

## 5. Conclusions

It was found that liquid phase sintering of a mixture of tin and atomized Al–10Zn alloy powders allows a ductile composite material with hardened aluminum matrix and tin inclusions located between the matrix grains to be prepared.

The sintered composite contains pores and areas of poor wetting with weak adhesive boundaries, which are mostly eliminated by subsequent pressing in a closed press mold at elevated temperatures. A result of the elimination of these defects is a significant increase in the yield strength and strain hardening rate of the (Al–10Zn)–40Sn composite at low deformations.

The wear intensity of the prepared (Al–10Zn)–40Sn composite is increased with increasing pressure on the friction surface under dry friction against steel. Its value depends on the sliding speed in a more complicated way and has an optimal value at *V* = 0.6 m/s.

The main wear mechanisms of the composites of Al–Sn system under the dry friction process are oxidative wear and a delamination of highly deformed subsurface grains of the aluminum matrix. The formed wear particles have high hardness and scratch the surface of a steel counterbody.

## Figures and Tables

**Table 1:** Chemical composition (wt.%) of the steel counterbody (Standard RF 4543).

C	Si	Mn	Ni	Cr	Cu	Fe
0.39	0.25	0.67	0.22	0.92	0.21	balance

**Table 2:** Effect of the sintering mode and subsequent *HP* on the porosity and mechanical properties of (Al–10Zn)–40Sn CM.

Properties	Sintering Mode				(600 °C; 1 h) + *HP*	
	570 °C; 1 h	585 °C; 1 h	600 °C; 1 h			
Porosity, %	14.2 ± 0.3	11.9 ± 0.3	5.6 ± 0.2	0.8 ± 0.1 *	1 ± 0.1	0.1 ± 0.1 *
σ_0.2_, MPa	30 ± 2	34 ± 4	44 ± 3	45 ± 4 *	95 ± 5	57 ± 5 *
σ_2_, MPa	38 ± 3	46 ± 5	59 ± 4	57 ± 5 *	118 ± 6	68 ± 5 *
σ_4_, MPa	38 ± 4	50 ± 4	64 ± 5	62 ± 3 *	121 ± 4	80 ± 3 *
σ_B_, MPa	39 ± 3	53 ± 4	77 ± 4	74 ± 4 *	129 ± 6	100 ± 5 *
Deformation before fracture, %	4.2 ± 0.2	10.8 ± 0.4	18 ± 0.4	16 ± 0.5 *	>20	>20 *

* The data were obtained for Al–40Sn composite.

**Table 3:** Effect of the *HP* processing and parameter *P·V* on the linear wear intensity *Ih* (µm/m) of (Al–10Zn)–40Sn CM under dry friction against steel. Sintering mode: 600 °C; 1 h.

Method of Preparing	*V* (m/s)	*P* (MPa)					
		1		3		5	
Sintering (*S*)	0.6	0.17 ± 0.02	0.15 ± 0.02 *	0.31 ± 0.03	0.24 ± 0.03 *	0.38 ± 0.04	0.38 ± 0.03 *
*S + HP*	0.6	0.12 ± 0.03	0.13 ± 0.02 *	0.17 ± 0.02	0.20 ± 0.03 *	0.25 ± 0.03	0.25 ± 0.03 *
*S + HP*	0.3	0.14 ± 0.02	0.18 ± 0.03 *	0.23 ± 0.03	0.30 ± 0.04 *	0.31 ± 0.02	0.34 ± 0.04 *
*S + HP*	0.9	0.12 ± 0.02	0.10 ± 0.02 *	0.17 ± 0.03	0.18 ± 0.02 *	0.33 ± 0.03	0.34 ± 0.03 *

* The data were obtained for Al–40Sn composite.

**Table 4:** Elemental composition (wt.%) of the friction surface of the CM samples. Sintering mode: 600 °C; 1 h.

Composite Sample	Method of Preparation	Friction Mode (*P*·*V*) [MPa·m/s]	Element				
			Oxygen	Iron	Tin	Zinc	Aluminum
(Al–10Zn)– 40Sn	Sintering (*S*)	5 · 0.6	34.7	16.4/32.7 *	20.1	1.4	Rest
	*S* + *HP*	5 · 0.3	37.4	6.1/15.8 *	21.2	2.6	Rest
		5 · 0.6	33.6	14.3/18.0 *	21.8	1.8	Rest
		5 · 0.9	28.0	21.4/36.9 *	21.4	2.3	Rest
Al–40Sn		5 · 0.3	38.2	7.0/11.0 *	24.1	-	Rest
		5 · 0.9	35.8	13.6/21.4 *	21.7	-	Rest

* Content in damaged areas (pits).

## Data Availability

Data sharing is not applicable.
